# Mental health professionals’ attitudes toward patients with PTSD and depression

**DOI:** 10.3402/ejpt.v6.28693

**Published:** 2015-10-26

**Authors:** Thomas Maier, Hanspeter Moergeli, Michaela Kohler, Giovanni E. Carraro, Ulrich Schnyder

**Affiliations:** 1 Psychiatric Services of the Canton St. Gallen-North, Wil, Switzerland; 2 Department of Psychiatry and Psychotherapy, University Hospital Zurich, University of Zurich, Zurich, Switzerland; 3 Psychiatric University Hospital, Ludwig-Maximilian-University Munich, Munich, Germany; 4 Private Practice, Locarno, Switzerland

**Keywords:** Expert attitudes, mental illness, stigma, PTSD, depression

## Abstract

**Background:**

To date, mental health professionals’ attitudes toward posttraumatic stress disorder (PTSD), compared to other psychiatric disorders such as schizophrenia or depression, have rarely been studied.

**Objective:**

We assessed mental health professionals’ attitudes toward patients with PTSD compared to patients suffering from depression.

**Method:**

Case vignettes of a patient with either PTSD or depression were presented to two samples of mental health professionals: attendees of a conference on posttraumatic stress (*N=*226) or of a lecture for psychiatry residents (*N=*112). Participants subsequently completed a questionnaire that assessed their attitude reactions to the presented case.

**Results:**

Participants showed similarly positive attitudes toward depression and PTSD. PTSD elicited a more favorable attitude with regard to prosocial reactions, estimated dependency, attributed responsibility, and interest in the case, particularly in mental health professionals specializing in psychotraumatology. Across diagnoses, higher age and longer professional experience were associated with more positive attitudes toward patients.

**Conclusions:**

Mental health professionals’ positive attitudes toward patients with depression and PTSD correlate with their specific knowledge about the disorder, their level of professional training, and their years of professional experience.

**Limitations:**

The instruments used, although based on established theoretical concepts in attitude research, were not validated in their present versions.

The attitude of the general public toward people suffering from mental disorders is often negative and stigmatizing because of their prevailing adverse stereotypes (Angermeyer & Dietrich, 2006; Angermeyer & Matschinger, 2005; Angermeyer, Matschinger, & Schomerus, 2013; Angermeyer et al., 2014; Brockington, Hall, Levings, & Murphy, 1993; Lauber, Nordt, Sartorius, Falcato, & Rössler, 2000; Link, 1987; Link, Phelan, Bresnahan, Stueve, & Pescosolido, 1999; Reavley, Mackinnon, Morgan, & Jorm, 2014; Wang & Lai, 2008; Weiner, Perry, & Magnusson, 1988; Yap, Mackinnon, Reavley, & Jorm, 2014). Although to date few studies have been published on the attitudes of mental health professionals toward their patients, a number have found similarly negative, stigmatizing trends (Aydin, Yigit, Inandi, & Kirpinar, 2003; Kopera et al., 2014; Lauber, Anthony, Ajdacic-Gross, & Rössler, 2004; Nordt, Rössler, & Lauber, 2006; Peris, Teachman, & Nosek, 2008; Reavley et al., 2014). A thorough understanding of professionals’ attitudes toward their patients is important not only because of the potential negative consequences for a sustainable, successful, therapeutic relationship, but also with regard to their role as opinion leaders.

The literature offers various theoretical explanatory models on the formation of negative attitudes toward people with mental disorders (cf. Yap et al., 2014). These models are applicable to the general public as well as to health professionals. Brockington et al. (1993) identified four stereotypes—“dangerous,” “incompetent,” “parasitic,” and “one's own fault”—as the central mediators of negative attitudes toward the mentally ill. According to Corrigan et al.'s (2003) model, dismissive behavior toward people suffering from mental disorders is seen as a consequence of perceived dangerousness and the attribution of responsibility. Angermeyer and Matschinger (2003) found a strong relationship between the appraisal of a mental illness and social distance. Perceived dangerousness and dependency determine feelings such as fear, antipathy, and anger. These feelings in turn are decisive for a person's desire for social distance.

Given the strong emphasis on an external cause as a *sine qua non* for the onset of the disorder (De Vries, 1996; Maier, 2006), posttraumatic stress disorder (PTSD) might elicit a different attitude than other mental disorders. However, although PTSD is reported to be highly prevalent—with an estimated lifetime prevalence of approximately 7% (Kessler, Sonnega, Bromet, Hughes, & Nelson, 1995)—attitudes toward patients suffering from PTSD have rarely been studied. A few studies have compared attitudes toward PTSD patients with attitudes toward patients suffering from other disorders (Reavley et al., 2014; Yap et al., 2014). Borderline personality disorder seems to generate a more negative attitude than PTSD (Calvert, 1996), and patients with bulimia are seen as more responsible for their condition than those suffering from PTSD (Hayes & Wall, 1998). Major depression evoked attitudes similar to those for PTSD (Arbanas, 2005; Reavley et al., 2014). Some of these studies, however, suffer from a number of methodological flaws. For instance, they assessed attitude using only one or two variables, although attitude is better understood as a multidimensional concept (Link, Yang, Phelan, & Collins, 2004). Additionally, in many of these studies, only a diagnostic label was presented to participants without any details about the traumatic event or psychopathological features. A study by Weiner et al. (1988) and another by Cubela (1997) demonstrated that background information about the nature of the condition of the PTSD patient is of essential importance for the emergence of a specific attitude toward the patient. Apparently, people not only form their attitudes based on global information such as the label “PTSD,” but they also integrate information such as the severity of the traumatic event or the patient's perceived responsibility for the event. Depending on this additional information, different attitudes can result in patients with the same diagnosis.

## Objective

We conducted a study evaluating the attitudes of mental health professionals, primarily psychiatrists, psychiatry residents, and clinical psychologists, toward patients suffering from PTSD as compared to patients with depression. We hypothesized that because of the external cause of their disease, PTSD patients would evoke more positive attitudes than depressive patients. Depression was chosen as a comparison because it is one of the most frequent mental disorders, and we presumed that all participating mental health professionals had experience in treating patients suffering from depression.

## Methods

### Sampling

We assessed attitudes in two samples of mental health professionals. One sample comprised mental health professionals specializing in psychotraumatology, and the second sample consisted of postgraduate students in their residencies who were training to become psychiatrists. The first sample was taken from the audience of a conference on psychotraumatology in Zurich. The second sample was recruited from attendees of a postgraduate lecture for psychiatry residents also held in Zurich. The procedures for data acquisition were identical in both samples: the study was integrated as an *ad hoc* presentation during a plenary session of the corresponding event. It was not preannounced to the audience, so the participants neither specifically attended nor avoided that particular session because of the study. Only once the presentation was underway were the attendees informed about the topic of attitude and invited to take part in a study described as an “evaluation of attitudes toward mental disorders among professionals.” Based on written material handed out to the audience, research questions were subsequently explained and displayed openly to the audience. The study was approved by the ethics committee of the canton of Zurich.

In the sample of professionals specializing in psychotraumatology, 248 questionnaires were distributed in the audience, of which 226 were returned (response rate 91.1%). In the sample of postgraduate students, 121 questionnaires were distributed, of which 112 were returned (92.6%). Table 1 shows the sociodemographic characteristics of both samples.

**Table 1:** Sociodemographic and professional characteristics of the two samples

	Mental health professionals specializing in psychotraumatology (*n*=226)	Residents specializing in general psychiatry (*n*=112)
Sex		
Female	147 (65.0%)	52 (46.4%)
Male	73 (32.3%)	59 (52.7%)
Missing	6 (2.3%)	1 (0.9%)
Age (years)	*M*=50.5 (SD=9.6) Range: 24–79	*M*=35.8 (SD=7.1) Range: 25–66
Professional education (multiple entries)		
Medicine	98 (43.4%)	108 (96.4%)
Psychology	92 (40.7%)	1 (0.9%)
Social work	15 (6.6%)	0 (0%)
Other education	28 (12.4%)	4 (3.6%)
Missing values	5 (2.2%)	0 (0%)
Years of experience with mentally ill patients		
0–4	27 (11.9%)	88 (78.6%)
5–9	31 (13.7%)	17 (15.2%)
10 or more	160 (70.8%)	7 (6.3%)
Missing values	8 (3.5%)	0 (0%)
Clinical experience with PTSD patients		
None	19 (8.4%)	28 (25.0%)
1–5 cases	48 (21.2%)	64 (57.1%)
6 or more cases	136 (60.2%)	19 (17.0%)
Missing values	23 (10.2%)	1 (0.9%)
Clinical experience with depressed patients		
None	14 (6.2%)	4 (3.6%)
1–5 cases	14 (6.2%)	14 (12.5%)
6 or more cases	178 (78.8%)	93 (83.0%)
Missing values	20 (8.8%)	1 (0.9%)

## Instruments

### Case vignettes

Case vignettes represent a standard component of attitude research (Link et al., 2004). A case vignette that described either a patient with a depressive disorder or a patient with PTSD was handed out randomly to participants. Both case vignettes were developed in analogy to Jorm et al.'s (2005) procedure. They both described a young woman who met diagnostic criteria for either depression or PTSD [in accordance with the Diagnostic and Statistical Manual of Mental Disorders 4th ed., text rev. (American Psychiatric Association, 2000)] but did not explicitly indicate a diagnosis. Both vignettes included the same number of psychopathological symptoms and were conclusive for either PTSD or depression. The patient's name, sex, and age were identical in both vignettes to avoid bias created by characteristics not related to the disorder. For better comparability, to reduce potential confounders, and given that more women than men suffer from PTSD and from depression (Bojanovsky & Stubbe, 1982; Breslau, Chilcoat, Kessler, Peterson, & Lucia, 1999; Kopera et al., 2014), we decided to use a female patient for both vignettes. In a previous pilot study, both vignettes were presented to 15 professional psychiatrists with extensive clinical experience. All 15 psychiatrists established the correct diagnosis based on the information provided in the case vignettes. The vignettes were presented in German, the participants’ native language. The translated version reads as follows:

#### Depression

Andrea M. is 35 years old. For 2 months now, she has been suffering from increased fatigue and has difficulties concentrating. She is constantly ruminating about her unhappy situation. Her mood is depressed, and she has lost interest in and pleasure for most of the things she liked to do in the past. She feels worthless and has a pessimistic outlook of the future. Sometimes, she is disgusted with her life, particularly at night, when she wakes up and cannot sleep anymore.

#### PTSD

Andrea M. is 35 years old. For 2 months now, she has been suffering from increased nervousness and has difficulties concentrating. She is extraordinarily jumpy and irritable. Again and again, thoughts about a distressing incident intrude into her mind. She is feeling numb and hardly able to participate in the normal activities of daily life. Apart from that, she has the feeling of a limited future. At night, she wakes up because of nightmares and finds it hard to fall asleep again.

### Questionnaires

We aimed to evaluate different components with regard to the participants’ attitude. Our goal was to assess the *attitudinal reactions* (emotional, cognitive, and behavioral) in accordance with well-known models of Angermeyer and Matschinger (2003), Corrigan et al. (2003), and Link and Phelan (2001). For some of the components with relevance to the participants’ attitudes, new items were designed by adapting similar preexisting scales (see below). We decided to use a multiple-choice format with five- and six-level Likert scales. The decision to use the multiple-choice format was made for several reasons: the questions are unequivocal, the answers require little time, and the results are easier to analyze.

The main questionnaire that assessed the attitudinal reactions contained 23 items. These questions aimed to assess the *prosocial reactions* provoked by the patient (three five-level Likert questions), the characteristic *stereotypes* toward psychiatric patients concerning *dependency/autonomy* (four five-level Likert questions), the *attribution of responsibility* (four five-level Likert questions), the *appraisal of the case* as interesting and motivating (four five-level Likert questions), *therapeutic alliance* (six five-level Likert questions), and *prognostic expectations with and without professional help* (one six-level Likert question each). All of these questions were based on several attitude scales published by Alexander and Luborsky (1986), Angermeyer, Buyantugs, Benzine, and Matschinger (2004, cf. also Angermeyer & Matschinger, 2004; Dreißing & Vogues, 2000), Brockington et al. (1993), Corrigan et al. (2003), Rössler, Salize, Trunck, and Vogues (1996), and Weiner (1986, 2000). The attitude scales comprised the sum of the corresponding items.

One auxiliary item concerned the identification of the disorder in question. This is a well-established method that was previously used by Star (1955) and later by Jorm et al. (1997), and Chung, Chen, Lam, Chen, and Chan (1997) to ensure that participants are giving their opinion on the disorder in question. Additionally, after completing the main questionnaire, participants gave details regarding their sociodemographic data and completed the Scale of Social Desirability by Stöber (1999) so that we were able to estimate the validity of their answers with regard to distortion through social desirability. This scale includes eight items (only yes/no answers) on social desirability.

### Procedures

After receiving general information about the study, all mental health professionals present in the audience during the session received a package that contained a written case vignette and a response sheet. Attendees also received written information noting that their participation was absolutely voluntary and that their return of the completed form was considered as informed consent to participate. The sheets in the package had an identical layout for all participants but were presorted in a way that participants randomly received either the depression vignette or the PTSD vignette. Participants were not informed that two different case vignettes were distributed among the audience. First, participants filled in their individual sociodemographic data. Subsequently, the 32 questions were presented to the audience using an LCD projector and read aloud by M. K. so that all participants could read and hear the questions simultaneously and respond immediately according to the case vignette they had been provided. The presentation took 19 min in total.

### Statistical analyses

All statistical analyses were performed using IBM SPSS Statistics (Version 22.0). The level of significance was set at *p*≤0.05. Descriptive statistics, Pearson chi-square, and *t*-tests were used to characterize both samples of participants and their diagnostic accuracy. Kolmogorov–Smirnov test indicated that the distribution of all attitude variables was non-normal (*p*<0.10). Thus, we tested our hypotheses with both non-parametric and parametric analyses. Because both analyses yielded identical results regarding significance, we decided to report parametric analyses only because they are easier to recognize. Accordingly, univariate ANOVAs were used to compare participants’ attitudes toward patients with depression versus PTSD in both samples [mental health professionals specializing in psychotraumatology (*N=*226) and psychiatry residents (*N=*112)]. To compare the attitudes between the two samples, univariate ANOVAs were also calculated (*N=*338). Finally, Pearson correlations provided information about the associations between the diagnostic condition, therapist variables, and attitudes toward patients in the aggregated sample.

## Results

### Sociodemographic and professional variables of the sample

The majority of the participating health professionals specializing in psychotraumatology were female (65%) and had an education as psychiatrists (43.4%) or psychologists (40.7%), whereas in the sample of psychiatry residents the proportion of females was smaller (46.4%; Pearson χ^2^=12.3, df=1, *p*<0.001). Given the framework of a postgraduate lecture for psychiatry residents, almost all participants in the postgraduate group had a medical education (96.4%; Pearson χ^2^=85.5, df=1, *p*<0.001). Participants in the psychotraumatology group were on average markedly older (50.5 years vs. 35.8 years; *t=*14.0, df=323, *p*<0.001) and consequently had more clinical experience than the postgraduate group. In particular, the first group was more experienced in the treatment of PTSD patients, whereas the two groups had similar experience in the treatment of patients with depression (cf. Table 1).

### Diagnostic accuracy

In the psychotraumatology group, 103 (88%) of the 117 participants correctly diagnosed depression, whereas 85 (78%) of the 109 participants correctly diagnosed PTSD according to the DSM-IV criteria. In the postgraduate group, 96.4% (54 out of 56) correctly diagnosed depression and 78.6% (44 out of 56) correctly diagnosed PTSD. The percentage of incorrect diagnoses was, in both groups, unexpectedly high. Therefore, we performed all analyses twice: with and without those participants who had diagnosed their case incorrectly. Both analyses revealed the same pattern when comparing diagnostic conditions and participant samples. Furthermore, the pattern of correlations remained stable with regard to the correlations between the therapist variables and attitudes. Therefore, we decided to report the results of all participants.

### Participant groups and attitudes

Participants’ attitudes toward both diagnoses were generally positive (Tables 2 and 3). In both groups, the attitudes toward depressed patients differed from the attitudes toward PTSD patients in some aspects: the participating health professionals attributed responsibility for the condition more often to patients with depression than to patients with PTSD (group 1: *F*=61.6; df=1, 217; *p*<0.001, group 2: *F*=13.8; df=1, 108; *p*<0.001). Furthermore, in both groups, the depression case was estimated to be less interesting than the PTSD case (Tables 2 and 3 specify statistical details). Only in the psychotraumatology group were patients with depression estimated to be more dependent than patients with PTSD. In the postgraduate group, the prosocial reaction toward the two different patient types was almost identical, whereas in the psychotraumatology group, the PTSD case evoked more positive reactions. Participants from the postgraduate sample expected the therapeutic relationship with the patient with PTSD to be more difficult than that with the patient with depression, and the prognosis for the patient with PTSD was also estimated to be poorer than that for the patient with depression. In the psychotraumatology group, no such difference was found. Across the two patient conditions, the psychotraumatology group showed a significantly more positive prosocial reaction than the psychiatry residents (cf. Table 4). Furthermore, the case estimation and the prognosis with professional help were significantly more positive in the psychotraumatology group.

**Table 2:** Attitudes toward patients with depression or PTSD in mental health professionals specializing in psychotraumatology (*N*=226)

	Diagnostic condition		ANOVA		
		
Attitude variable	Depression (*n*=117) *M* (SD)	PTSD (*n*=109) *M* (SD)	*F*	df	*p*
Prosocial reaction toward patient	10.17 (1.97)	11.13 (1.95)	13.35	1, 200	<0.001
Stereotypes; patient estimated as					
Dependent	5.37 (1.73)	4.63 (1.80)	9.57	1, 213	<0.01
Autonomous	4.23 (1.62)	4.79 (1.88)	5.65	1, 217	<0.05
Responsibility attributed	10.46 (2.00)	13.02 (2.59)	61.59	1, 196	<0.001
Case appraisal	13.24 (3.02)	15.70 (2.70)	39.97	1, 216	<0.001
Expected quality of therapeutic relationship	21.56 (3.52)	21.74 (3.32)	0.14	1, 200	ns
Prognosis with professional help	1.95 (0.89)	2.13 (0.98)	1.78	1, 213	ns
Prognosis without professional help	4.53 (1.14)	4.87 (1.08)	5.11	1, 209	<0.05

Maximum range of the sum scores: prosocial reaction 3–15 (3=not at all likeable; 15=very likeable); dependency 2–10 (2=not at all dependent; 10=very dependent); autonomy 2–10 (2=not at all autonomous; 10=very autonomous); responsibility 4–20 (4=one's own fault; 20=guilt of others); case appraisal 4–20 (4=not at all interesting; 20=very interesting); therapeutic relationship 6–30 (6=very poor relationship; 30=very good relationship); prognosis 1–6 (1=good prognosis; 6=poor prognosis).

**Table 3:** Attitudes toward patients with depression or PTSD in psychiatry residents (*N*=112)

	Diagnostic condition		ANOVA		
		
Attitude variable	Depression (*n*=56) *M* (SD)	PTSD (*n*=56) *M* (SD)	*F*	df	*p*
Prosocial reaction toward patient	9.52 (1.58)	9.50 (1.90)	0.003	1, 108	ns
Stereotypes; patient estimated as					
Dependent	5.16 (1.75)	5.18 (1.61)	0.003	1, 110	ns
Autonomous	4.20 (1.22)	4.50 (1.60)	1.23	1, 109	ns
Responsibility attributed	10.43 (1.88)	12.02 (2.57)	13.78	1, 108	<0.001
Case appraisal	11.75 (2.77)	13.20 (3.22)	6.50	1, 110	<0.05
Expected quality of therapeutic relationship	21.55 (2.76)	20.23 (2.69)	6.31	1, 106	<0.05
Prognosis with professional help	2.11 (0.68)	2.48 (0.99)	5.46	1, 110	<0.05
Prognosis without professional help	4.57 (1.16)	4.84 (1.10)	1.52	1, 109	ns

Maximum range of the sum scores: prosocial reaction 3–15 (3=not at all likeable; 15=very likeable); dependency 2–10 (2=not at all dependent; 10=very dependent); autonomy 2–10 (2=not at all autonomous; 10=very autonomous); responsibility 4–20 (4=one's own fault; 20=guilt of others); case appraisal 4–20 (4=not at all interesting; 20=very interesting); therapeutic relationship 6–30 (6=very poor relationship; 30=very good relationship); prognosis 1–6 (1=good prognosis; 6=poor prognosis).

**Table 4:** Differences in attitudes between professionals specializing in psychotraumatology and psychiatry residents (*N*=338)

	Group of participants		ANOVA		
		
Attitude variable	Psychotraumatology group (*n*=226) *M* (SD)	Psychiatry residents (*n*=112) *M* (SD)	*F*	df	*p*
Prosocial reaction toward patient	10.6 (2.01)	9.5 (1.74)	24.715	1, 330	<0.001
Stereotypes; patient estimated as					
Dependent	5.0 (1.80)	5.2 (1.67)	0.614	1, 325	ns
Autonomous	4.5 (1.77)	4.4 (1.42)	0.537	1, 328	ns
Responsibility attributed	11.7 (2.62)	11.2 (2.37)	2.249	1, 306	ns
Case appraisal	14.4 (3.12)	12.5 (3.08)	29.622	1, 328	<0.001
Expected quality of therapeutic relationship	21.6 (3.42)	20.9 (2.79)	3.782	1, 308	ns
Prognosis with professional help	2.0 (0.94)	2.3 (0.87)	5.853	1, 325	<0.05
Prognosis without professional help	4.7 (1.12)	4.7 (1.13)	0.002	1, 320	ns

Maximum range of the sum scores: prosocial reaction 3–15 (3=not at all likeable; 15=very likeable); dependency 2–10 (2=not at all dependent; 10=very dependent); autonomy 2–10 (2=not at all autonomous; 10=very autonomous); responsibility 4–20 (4=one's own fault; 20=guilt of others); case appraisal 4–20 (4=not at all interesting; 20=very interesting); therapeutic relationship 6–30 (6=very poor relationship; 30=very good relationship); prognosis 1–6 (1=good prognosis; 6=poor prognosis).

### Associations between participants’ variables and attitudes

At first, in the aggregated sample of all participants, the diagnostic condition (depression vs. PTSD) was tested for correlations with attitudes toward patients (Table 5). Across the two participant groups, PTSD provoked more positive prosocial reactions than depression (*r=*0.15, *p*<0.01). Patients with depression were estimated to be more responsible for their condition than patients with PTSD (Table 5 specifies statistical details). Furthermore, the PTSD case was considered markedly more interesting by the participating mental health professionals. However, the prognosis was estimated to be slightly more favorable in the depression case. The age of participants correlated with the prosocial reactions, with the case estimation, and expected quality of the therapeutic relationship. As the participants’ age increased, their prosocial reactions were stronger, they estimated the case to be more interesting, and they expected the therapeutic relationship to be better. In accordance with these findings, the years of clinical experience also correlated with a more positive prosocial reaction toward patients, a more positive case estimation, and a better therapeutic relationship. Only weak correlations were found with regard to the social desirability of participants’ answers. Participants’ sex and type of education (medical vs. non-medical) were not correlated with any of the attitude variables.

**Table 5:** Correlations between therapist variables and attitudes toward patients (*N*=338)

	Prosocial reactions	Dependency	Autonomy	Responsibility	Case appraisal	Therapeutic relationship	Prognosis with help	Prognosis without help
Diagnostic condition	0.15**	–0.14*	0.14**	0.43***	0.32***	–0.05	0.13*	0.14*
Age of participant	0.29***	–0.04	–0.04	0.08	0.23***	0.19***	–0.02	–0.03
Female sex of participant	0.10	–0.02	–0.01	–0.01	0.10	–0.08	–0.01	0.03
Social desirability	0.12*	–0.02	–0.09	–0.11*	0.07	0.04	0.00	0.05
Years of clinical experience	0.28***	0.00	0.01	0.11	0.24***	0.22***	–0.12*	0.01
Medical education	–0.06	–0.04	0.00	0.09	–0.09	0.05	0.09	–0.05

Pearson correlations. Diagnostic condition: 1=depression; 2=PTSD. Social desirability: 0–8 (0=very low; 8=very high). Medical education: 0=no (other education); 1=yes. For attitude variables, cf. Table 4.

* *p*≤0.05

** *p*≤0.01

*** *p*≤0.001.

## Discussion

This study assessed the attitudes of mental health professionals toward depression and PTSD. Emotional, cognitive, and behavioral attitudinal reactions of participants were evaluated through a series of questions, case vignettes were used instead of only diagnostic labels, and mental health professionals at different levels of their education were recruited for participation. Based on these methodological characteristics, our findings may represent a valid estimation of the prevailing attitudes of mental health professionals toward these two diagnostic conditions as compared to previous studies on attitudes. As the study was conducted without mentioning specific diagnostic entities to participants and as the case vignettes used did not explicitly mention diagnoses, our results may reflect the immediate attitudinal reactions of mental health professionals toward specific clinical situations. The fact that a considerable number of participants (*N*=52) failed to establish the correct diagnosis may be a consequence of this avoidance of labeling.

The results of our study demonstrate that the attitudes of mental health professionals toward a patient with PTSD are at least similarly positive as those toward a patient with depression. In some categories of attitudes (prosocial reactions, dependency/autonomy, attribution of responsibility, and interest for the case), reactions toward patients with PTSD patients were even more positive than reactions toward patients with depression. Overall, positive responses toward both vignettes were far more in evidence than negative responses, indicating there was a good level of comprehension and understanding toward the two patient conditions presented. This generally positive attitude may be seen as an expression of professionalism in mental health workers. PTSD has the potential to attract even more positive attitudes as it is perceived by many to be caused predominantly by an external event. In our sample, this effect increased in line with the length of the mental health professionals’ experience. Our results are consistent with other findings comparing PTSD and other psychiatric disorders (Nordt et al., 2006; Reavley et al., 2014; Yap et al., 2014). The attitudinal reactions toward PTSD are at least as positive as attitudes toward depression, which is in line with the results of Arbanas (2005) and Reavley et al. (2014).

Apart from the disorder condition, some therapist variables were of relevant influence: higher age and, in conjunction with that, longer professional experience were correlated with markedly more positive attitudes in both diagnostic conditions. These results are consistent with preexisting results in the literature. Age and years of professional experience are variables that were identified as relevant additional factors in determining a more positive attitudinal reaction in other studies (Angermeyer et al., 2014; Caldwell & Jorm, 2001; Lauber, Nordt, Braunschweig, & Rössler, 2006; Peris et al., 2008). These findings may indicate that knowledge about a specific disorder, interest in a disorder, and personal experience with patients suffering from that specific disorder are factors that positively influence attitudes toward patients. It can be hypothesized that positive attitudes toward patients suffering from mental disorders are not preexisting, but rather result from specific knowledge and personal experience (cf. Peris et al., 2008; Wang & Lai, 2008).

## Conclusions

Even in mental health professionals, some diagnostic conditions appear to elicit more positive attitudinal reactions than others. More positive attitudes correlate with greater clinical experience and with specific knowledge of and education about mental disorders. It can be hypothesized that positive attitudinal reactions are—also in mental health professionals—not necessarily preexisting, but have to be developed through continuous professional education. In turn, negative attitudes toward mentally ill persons may not necessarily result from conscious judgments but may represent the mostly spontaneous, intuitive reactions of humans. This effect can be demonstrated even in mental health professionals (Kopera et al., 2014). The capacity of mental health professionals to approach their patients in an unprejudiced manner must be developed through careful education and continuous professional training. This situation highlights the importance of a continuous reflection on the attitudes health professionals have toward patients because they act as opinion leaders in the general public (Angermeyer et al., 2014).

## Limitations

There are a number of limitations that stem from the explorative character of the study. We examined the self-report of health professionals about *in sensu* attitudes and did not directly observe their behavior. Although this method is well established and the influence of the participants’ social desirability on their answers was negligible, our results can be understood only as an approximation of true interpersonal behavior. In this vein, we should also mention the special character of case vignettes. Vignettes are one of the preferred methods in attitude research, but they can never provide all relevant details in a real therapeutic setting. Furthermore, our sampling method was selective and not representative for mental health professionals in general. Finally, the instruments we used, although based on established theoretical concepts in attitude research, were not validated in their present versions.

## Supplementary Material

Mental health professionals’ attitudes toward patients with PTSD and depression
