# Supplementary material for: Mental health professionals’ attitudes toward patients with PTSD and depression
Source: Eur J Psychotraumatol. 2015 Oct 26;6:10.3402/ejpt.v6.28693. doi: 10.3402/ejpt.v6.28693 (PMC4623286; doi:10.3402/ejpt.v6.28693)
Supplement: Mental health professionals’ attitudes toward patients with PTSD and depression [file EJPT-6-28693-s001.pdf]

Supplemental material

Questionnaire (32 Questions)

Dear attendees of this conference

Thank you for participating in this study. Please take your handout and proceed to page two, where you will find a case vignette of a clinical case. Please read it carefully. Imagine that it informs you about a new case referred to you.

Please try to visualize Andrea M. in the most live manner. If you don't have a clear imagination of Andrea M. yet, you better take another moment. This is important, because all the following questions relate to Andrea M.

Questions will be displayed on the screen according to the numbers of the questionnaire and read out. Please try to answer all the questions by ticking the best fitting box.

(Identification of the disorder)

Question 1) Based on the information given so far, which of the following diagnoses you consider to be the most probable? Please tick the box with the most appropriate diagnosis!

Question 1				
❍ Schizophrenia, undifferentiated type	❍ PTSD	❍ Generalized anxiety disorder	❍ Major depression	❍ None of the diagnoses mentioned

(Prosocial reactions)

Question 2) Now we would like to ask you about your feelings when you think of Andrea M. and how intense these feelings are.

2.1) I feel touched.
2.2) I feel sympathy for Andrea M.
2.3) I have the wish to help Andrea M.

Question 2.1	❍ not at all	❍ a little	❍ Moderate	❍ quite	❍ very much
Question 2.2	❍ not at all	❍ a little	❍ moderate	❍ quite	❍ very much
Question 2.3	❍ not at all	❍ a little	❍ moderate	❍ quite	❍ very much

(Stereotypes concerning dependency/autonomy)

Question 3) Now we propose you some characteristics of Andrea M. According to you, to what extent these characteristics are applicable to Andrea M.?

3.1) Dependent
3.2) Determined
3.3) Clinging
3.4) Independent

Question 3.1	❍ not applicable	❍ applicable a little	❍ partly applicable	❍ quite applicable	❍ very much applicable
Question 3.2	❍ not applicable	❍ applicable a little	❍ partly applicable	❍ quite applicable	❍ very much applicable
Question 3.3	❍ not applicable	❍ applicable a little	❍ partly applicable	❍ quite applicable	❍ very much applicable
Question 3.4	❍ not applicable	❍ applicable a little	❍ partly applicable	❍ quite applicable	❍ very much applicable

(Attribution of responsibility)

Question 4) The following questions are about responsibility for the suffering of Andrea M. To what extent you think the following statements are appropriate?

4.1) Other people are mainly responsible for the suffering Andrea M.
4.2) The main cause for Andrea M.'s suffering is up to herself.
4.3) Andrea M.'s suffering is a matter of fate.
4.4) Andrea M. is ill, because she can't cope well with crises and stressful events.

Question 4.1	❍ not applicable	❍ applicable a little	❍ partly applicable	❍ quite applicable	❍ very much applicable
Question 4.2	❍ not applicable	❍ applicable a little	❍ partly applicable	❍ quite applicable	❍ very much applicable
Question 4.3	❍ not applicable	❍ applicable a little	❍ partly applicable	❍ quite applicable	❍ very much applicable
Question 4.4	❍ not applicable	❍ applicable a little	❍ partly applicable	❍ quite applicable	❍ very much applicable

(Appraisal of the case)

Question 5) Now we would like to ask you to what extent you think some characteristics are applicable to Andrea M.'s case.

Andrea M.'s case is…
5.1) ... exciting.
5.2) ... usual
5.3) ... motivating
5.4) ... boring.

Question 5.1	❍ not applicable	❍ applicable a little	❍ partly applicable	❍ quite applicable	❍ very much applicable
Question 5.2	❍ not applicable	❍ applicable a little	❍ partly applicable	❍ quite applicable	❍ very much applicable
Question 5.3	❍ not applicable	❍ applicable a little	❍ partly applicable	❍ quite applicable	❍ very much applicable
Question 5.4	❍ not applicable	❍ applicable a little	❍ partly applicable	❍ quite applicable	❍ very much applicable

(Therapeutic alliance)

Question 6) Now imagine you take over the treatment of Andrea M. What kind of expectations you have concerning the therapeutic alliance to Andrea M.?

6.1) I will be able to help Andrea M.
6.2) Andrea M. will feel understood by me.
6.3) Andrea M. will rely on me.
6.4) Andrea M. will trust me that I commit myself to the attainment of her goals.
6.5) Andrea M. will collaborate with me seriosuly.
6.6) Andrea M.'s view and appraisal of her problem will be similar to mine.

Question 6.1	❍ not applicable	❍ applicable a little	❍ partly applicable	❍ quite applicable	❍ very much applicable
Question 6.2	❍ not applicable	❍ applicable a little	❍ partly applicable	❍ quite applicable	❍ very much applicable
Question 6.3	❍ not applicable	❍ applicable a little	❍ partly applicable	❍ quite applicable	❍ very much applicable
	❍	❍	❍	❍	❍

Question 6.4	not applicable	applicable a little	partly applicable	quite applicable	very much applicable
Question 6.5	❍ not applicable	❍ applicable a little	❍ partly applicable	❍ quite applicable	❍ very much applicable
Question 6.6	❍ not applicable	❍ applicable a little	❍ partly applicable	❍ quite applicable	❍ very much applicable

(Prognostic expectations with and without professional help)

Question 7) The following questions are about your estimation of Andrea M.'s prognosis.

7.1) What is your estimation of Andrea M.'s prognosis for the next 1-2 years in case she gets <u>adequate professional help</u> now?
7.2) What is your estimation of Andrea M.'s prognosis for the next 1-2 years in case she gets <u>no adequate professional help</u> now?

Question 7.1					
❍ Stable and complete remission	❍ Complete remission with possibility of relapse	❍ Partial remission	❍ Partial remission with possibility of relapse	❍ No improvement	❍ Further deterioration
Question 7.2					
❍ Stable and complete remission	❍ Complete remission with possibility of relapse	❍ Partial remission	❍ Partial remission with possibility of relapse	❍ No improvement	❍ Further deterioration

(Social desirability)

Question 8) Finally we would like to ask you some questions about yourself. We present you a number of statements to which anyone can agree to a varying extent. Please indicate to what extent each statement applies to yourself (not to Andrea M.!).

8.1) Sometimes I am late when I go to work or when I have a date.
8.2) In my life I always attained the goals I aimed for.
8.3) I judge other people only when I know all the facts.
8.4) It happened that I took advantage of someone.
8.5) I am never upset, when I am asked for a favor.
8.6) Sometimes I talk about things I don't understand.

8.7) I never intentionally said something that could have hurt someone else's feelings.
8.8) No matter to whom I talk, I am always a good listener.

Question 8.1	❍ Yes	❍ no
Question 8.2	❍ yes	❍ no
Question 8.3	❍ yes	❍ no
Question 8.4	❍ yes	❍ no
Question 8.5	❍ yes	❍ no
Question 8.6	❍ yes	❍ no
Question 8.7	❍ yes	❍ no
Question 8.8	❍ yes	❍ no
